# Extremes of baseline cognitive function determine the severity of delirium: a population study

**DOI:** 10.1093/brain/awad062

**Published:** 2023-02-28

**Authors:** Alex Tsui, Natalie Yeo, Samuel D Searle, Helen Bowden, Katrin Hoffmann, Joanne Hornby, Arley Goslett, Maryse Weston-Clarke, David Lanham, Patrick Hogan, Anna Seeley, Mark Rawle, Nish Chaturvedi, Elizabeth L Sampson, Kenneth Rockwood, Colm Cunningham, E Wesley Ely, Sarah J Richardson, Carol Brayne, Graciela Muniz Terrera, Zoë Tieges, Alasdair M J MacLullich, Daniel Davis

**Affiliations:** MRC Unit for Lifelong Health and Ageing at UCL, London, WC1E 7HB, UK; MRC Unit for Lifelong Health and Ageing at UCL, London, WC1E 7HB, UK; MRC Unit for Lifelong Health and Ageing at UCL, London, WC1E 7HB, UK; Geriatric Medicine, Dalhousie University, Halifax, NS B3H 2E1, Canada; MRC Unit for Lifelong Health and Ageing at UCL, London, WC1E 7HB, UK; MRC Unit for Lifelong Health and Ageing at UCL, London, WC1E 7HB, UK; MRC Unit for Lifelong Health and Ageing at UCL, London, WC1E 7HB, UK; MRC Unit for Lifelong Health and Ageing at UCL, London, WC1E 7HB, UK; MRC Unit for Lifelong Health and Ageing at UCL, London, WC1E 7HB, UK; MRC Unit for Lifelong Health and Ageing at UCL, London, WC1E 7HB, UK; MRC Unit for Lifelong Health and Ageing at UCL, London, WC1E 7HB, UK; MRC Unit for Lifelong Health and Ageing at UCL, London, WC1E 7HB, UK; Nuffield Department of Primary Care, University of Oxford, Oxford, OX2 6GG, UK; MRC Unit for Lifelong Health and Ageing at UCL, London, WC1E 7HB, UK; MRC Unit for Lifelong Health and Ageing at UCL, London, WC1E 7HB, UK; Marie Curie Palliative Care Research Department, UCL, London, W1T 7NF, UK; MRC Unit for Lifelong Health and Ageing at UCL, London, WC1E 7HB, UK; Geriatric Medicine, Dalhousie University, Halifax, NS B3H 2E1, Canada; School of Biochemistry & Immunology, Trinity Biomedical Sciences Institute, Dublin 2, Republic of Ireland; Department of Medicine, Vanderbilt University Medical Center, Nashville, TN, USA; AGE Research Group, Translational and Clinical Research Institute, Newcastle University, UK; Department of Public Health and Primary Care, University of Cambridge, UK; Edinburgh Dementia Prevention, University of Edinburgh, UK; Geriatric Medicine, Edinburgh Delirium Research Group, Usher Institute, University of Edinburgh, UK; SMART Technology Centre, Glasgow Caledonian University, Glasgow, UK; Geriatric Medicine, Edinburgh Delirium Research Group, Usher Institute, University of Edinburgh, UK; MRC Unit for Lifelong Health and Ageing at UCL, London, WC1E 7HB, UK

**Keywords:** delirium, baseline cognitive function, epidemiology

## Abstract

Although delirium is a significant clinical and public health problem, little is understood about how specific vulnerabilities underlie the severity of its presentation. Our objective was to quantify the relationship between baseline cognition and subsequent delirium severity.

We prospectively investigated a population-representative sample of 1510 individuals aged ≥70 years, of whom 209 (13.6%) were hospitalized across 371 episodes (1999 person-days assessment). Baseline cognitive function was assessed using the modified Telephone Interview for Cognitive Status, supplemented by verbal fluency measures. We estimated the relationship between baseline cognition and delirium severity [Memorial Delirium Assessment Scale (MDAS)] and abnormal arousal (Observational Scale of Level of Arousal), adjusted by age, sex, frailty and illness severity. We conducted further analyses examining presentations to specific hospital settings and common precipitating aetiologies.

The median time from baseline cognitive assessment to admission was 289 days (interquartile range 130 to 47 days). In admitted patients, delirium was present on at least 1 day in 45% of admission episodes. The average number of days with delirium (consecutively positive assessments) was 3.9 days. Elective admissions accounted for 88 bed days (4.4%). In emergency (but not elective) admissions, we found a non-linear U-shaped relationship between baseline global cognition and delirium severity using restricted cubic splines. Participants with baseline cognition 2 standard deviations below average (*z*-score = −2) had a mean MDAS score of 14 points (95% CI 10 to 19). Similarly, those with baseline cognition *z*-score = + 2 had a mean MDAS score of 7.9 points (95% CI 4.9 to 11). Individuals with average baseline cognition had the lowest MDAS scores. The association between baseline cognition and abnormal arousal followed a comparable pattern. C-reactive protein ≥20 mg/l and serum sodium <125 mM/l were associated with more severe delirium.

Baseline cognition is a critical determinant of the severity of delirium and associated changes in arousal. Emergency admissions with lowest and highest baseline cognition who develop delirium should receive enhanced clinical attention.

## Teaser

In a prospective study of older adults with unscheduled hospital admissions, Tsui *et al*. observe a U-shaped relationship between baseline global cognition—assessed by previous telephone interview—and the severity of episodes of delirium.

See Lennertz and Sanders (https://doi.org/10.1093/brain/awad115) for a scientific commentary on this article.

## Introduction

Delirium is a severe neuropsychiatric syndrome characterized by acute changes in arousal, inattention and other mental status changes. Its clinical importance is well-established: it affects one in four older inpatients, and in multiple settings, delirium is associated with adverse outcomes such as mortality, inpatient falls, delayed discharges and significant patient and carer distress.^1–5^ Delirium is also associated with future cognitive impairment and incident dementia.^6,7^ There is wide variability in the natural history of delirium.^8^ Although we know that older age, previous cognitive impairment and frailty are delirium risk factors,^9,10^ the combination of baseline cognition and acute illness could result in different degrees of delirium symptomatology. The influence of baseline cognition on subsequent delirium phenomenology has not been considered comprehensively. Yet, an empirical understanding of this relationship could affect delirium detection, assessment and management because the clinical significance of delirium symptoms might have different implications if framed in the context of a known baseline cognitive state.

Existing studies linking baseline cognitive function to delirium severity have used the methodological advantage of prospective follow-up in elective surgical populations.^11–13^ However, most delirium in secondary care presents in unselected unscheduled medical admissions with a much greater range of pre-existing cognitive impairment and frailty.^14^ Previous work in acute medical patients has assessed baseline cognition in two ways. First, by establishing a binary dementia diagnosis, or second by using cognitive testing on admission only in patients initially without delirium.^15,16^ This is a crucial issue because around two-thirds of delirium is present on admission.^15,17^ Very few reports in medical patients have assessed delirium severity in relation to baseline cognition.^18–20^ More broadly, we do not fully understand the overlap between delirium severity and arousal changes.^21^ Abnormal arousal may be a key driver to mortality after delirium, although its detailed quantification is under-represented in many delirium severity scales.^22^ Finally, we know little about the specific aetiological precipitants that might be associated with more severe delirium in general hospital settings.

To understand the influence of baseline cognition on delirium phenomenology (including abnormal arousal) across the whole spectrum of hospital presentations, we needed to characterize cognitive function in a stable community sample and then at each subsequent acute hospitalization systematically: (i) assess the severity of delirium on each day; (ii) relate this to baseline cognitive function; and (iii) understand the relationship between hospital setting, aetiological factors and delirium risk. We hypothesized that lower baseline cognition would lead to greater severity of delirium symptoms in the event of acute hospitalization.

## Materials and methods

### Population

**Figure 1:**
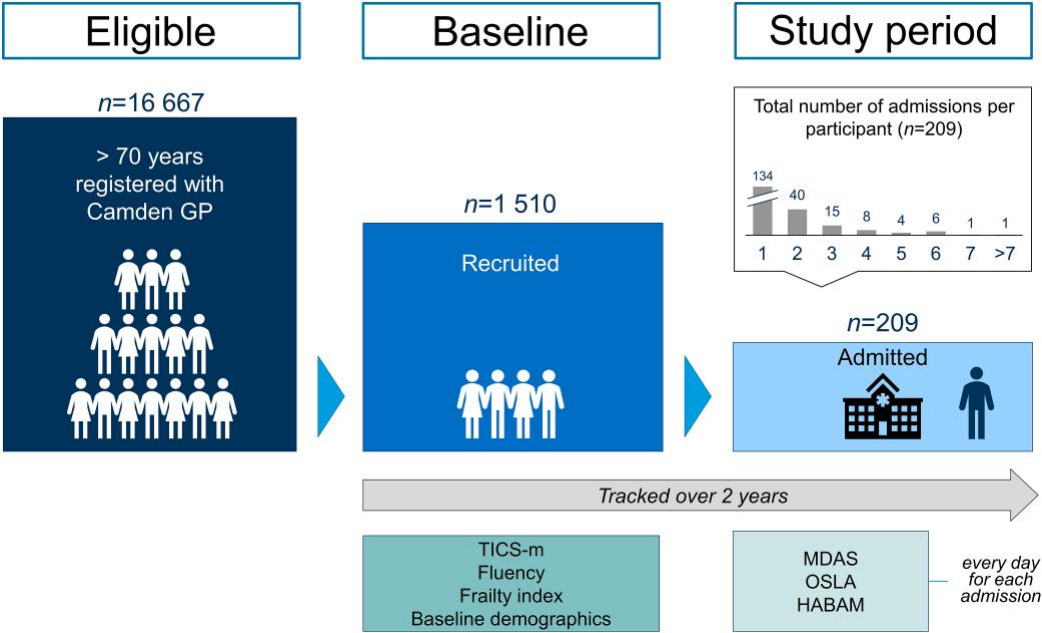
**Participant flow diagram.** Cohort structure showing sample and schedule of assessments.

The Delirium and Population Health Informatics Cohort is a prospective longitudinal population-representative sample of older adults aged ≥70 years in the London Borough of Camden, a central city region with a population of 260 000 residents (Fig. 1).^7,23^ The National Health Service in England provides >95% of healthcare, and Camden is served by a single primary care system (the Camden Clinical Commissioning Group representing 39 general practices) and two acute hospitals (University College Hospital, Royal Free Hospital). This report is a planned analysis of the participants recruited between January 2017 and December 2018. Our overall prespecified power calculations were for a separate outcome: a 2-year change in cognition at follow-up in the whole cohort. We anticipated that a minimum of 11% of the cohort would need to be admitted to provide meaningful estimates describing the relationship between baseline cognition and incident delirium.^23^

Eligible participants were aged ≥70 years and registered with a Camden-based general practitioner. Based on the coded problems in the primary care record, we did not approach those with severe hearing impairment, aphasia, or who could not speak English sufficiently to undertake any basic cognitive assessment or were in the terminal phase of illness. In addition to the primary care lists, we over-sampled from memory clinics and patients recently discharged from secondary care in an 8:1:1 ratio. Invitations were sent by letter. All individuals, or their nominated proxies, gave consent to participate. The direct sampling from memory clinics facilitated the inclusion of participants with pre-existing cognitive impairments and dementia.

### Baseline assessments

Most participants were assessed through telephone interviews. However, we enabled eligible participants with previously unidentified yet significant hearing impairment to be assessed at a home visit. Cognitive function was the primary measure, assessed using the modified Telephone Interview for Cognitive Status (TICS-m), which covers orientation, attention, naming, praxis, calculation and immediate and delayed recall of a 10-item non-semantically related word list.^24^ We supplemented this with the two verbal fluency tasks (generating words beginning with the same letter, number of animals) from the Addenbrooke’s Cognitive Examination to improve the measurement of executive function in the battery.^24,25^ Through interview and real-time access to all health and social care records through the Camden Integrated Digital Record, we assessed the following domains: socio-demographic factors, index of multiple deprivation, general health, co-morbidities, medications, health behaviours, hearing, vision, quality of life, dental health, continence, falls, depression, personal and instrumental activities of daily living. Frailty was quantified using a frailty index, representing the proportion of accumulated health deficits (0 to 1). This was derived using 28 items drawn from the baseline assessment and calculated in line with standard procedures.^26^ However, we did not include cognitive items to avoid collinearity with the primary cognitive measure. Further details for ascertaining baseline covariates have previously been published.^23^ For these analyses, we also checked to assess the extent to which inclusion or exclusion of specific frailty index items might have affected the fundamental relationships between frailty and delirium and found the index to be robust (Supplementary Table 1).

### Hospital assessments

All participants admitted to either of the acute hospitals were automatically flagged through daily electronic alerts and reviewed in person each day (Monday to Friday) from the day of admission by a researcher. We did not assess participants presenting to the emergency department who were discharged from there. At each assessment, we evaluated participants for changes in cognitive or physical function using the Memorial Delirium Assessment Scale (MDAS), Observational Scale of Level of Arousal (OSLA) and the Hierarchical Assessment of Balance and Mobility (although this last measure does not form part of this analysis).^21,27,28^ We recorded additional information on acute aetiology, medications, illness severity (NEWS) and laboratory findings. The NEWS integrates clinically abnormal physiological indices (heart rate, blood pressure, respiratory rate, oxygen saturations, supplemental oxygen requirements, alertness) giving a score from 0 to 20.^29,30^ Higher scores indicate risk of immediate deterioration, with scores above 4 indicating need for clinical review for escalation of care. Although NEWS2, which includes a measure of confusion, was introduced over the course of the study, this component had not been reliably implemented in routine care.^30^

### Ascertainment of delirium

We used the Diagnostic and Statistical Manual of Mental Disorders (DSM) IV criteria as the case ascertainment for the primary outcome because it is the most widely used definition and allows comparative estimates with other studies. Delirium was ascertained for every day of hospital admission using all available information. Complete interview questions are available on the Dementias Platform UK. On each day, we determined delirium to be present if individuals met criteria A (disturbance of consciousness), B (change in cognition and/or perception) and C (acute and fluctuates). By virtue of their inpatient admission, all participants were deemed to fulfil Criterion D (physiological consequence of a general medical condition).

### Statistical analyses

#### Outcome measures

##### Delirium severity

MDAS assesses 10 domains of delirium symptoms (awareness, orientation, short-term memory, digit span, attention capacity, disorganized thinking, perceptual disturbance, delusions, psychomotor activity, sleep–wake cycle, each scored out of 3) to give a 30-point measure of delirium severity. Abnormal arousal: the OSLA was designed to quantify grades of arousal changes in delirium, specifically quantifying eye opening, eye contact, posture and movement.^21^ It has 15 points, with higher scores representing deviations in arousal level in either direction, i.e. hyperactive or hypoactive.

#### Exposures

##### Baseline cognition

For the composite cognitive score, the TICS-m was scored out of 53 points, verbal fluency scored out of 14 points, summed to 67 points and standardized as a *z*-score (score-mean)/standard deviation. In the hospital setting, we separately examined elective and emergency admissions, as well as those presenting to surgical and internal medicine services. For the aetiology, we explored possible effects of broad aetiological categories based on laboratory results: C-reactive protein (≥20 mg/l); white cell count (<4 × 10^9^ cells/l; 4–11 × 10^9^ cells/l; ≥ 11 × 10^9^ cells/l); acute kidney injury (defined by the NHS England patient safety alert algorithm^31^); anaemia (haemoglobin <100 g/l) and hyponatraemia (<125 mM; 125–135 mM; 135–145 mM; ≥ 145 mM).

Frailty was quantified using a frailty index, as described before. Education was categorized as: up to primary (6 years of school); up to secondary (12 years school) and degree level or above. We also adjusted for time from baseline assessment to first admission to account for any possible interval change in cognition.

#### Missing data

Whole assessments that were missing due to falling on a weekend or public holiday (missing at random) were forwards-filled (Friday carried to Saturday) and backwards-filled (Sunday carried from Monday) in 24-h intervals for up to 4 days. Imputation is primarily a statistical technique. However, for backwards filling, this approach has the advantage of automatically carrying over information from the next working day’s chart review. Otherwise, data were assumed to be missing at random.

#### Models

In exploratory analyses, we examined the distribution of MDAS and OSLA scores by tertiles of baseline cognition (Supplementary Fig. 1), which suggested the underlying relationships might be non-linear. We investigated this by fitting restricted cubic splines with three knots. We used the default knot positioning from the Stata *mkspline* function, which operationalizes Harrell’s recommended percentiles with the additional restriction that the first and last knots are bound by the fifth-smallest and fifth-largest values of baseline cognition, respectively.^32^ We found equivalent results using fractional polynomials, a complementary technique for describing non-linear relationships (Supplementary Table 2 and Supplementary Fig. 2).

Models were estimated for each admission in each individual using mixed-effects linear regression, where each day’s MDAS or OSLA scores were the dependent variable, adjusted by age, sex, baseline cognition [standardized as (score-mean)/standard deviation], frailty index, NEWS and time from baseline assessment to first admission.

##### Sensitivity analysis

Because MDAS items 2 (disorientation) and 3 (short-term memory impairment) may be higher because of previous cognitive impairment (i.e. worse baseline cognitive scores), we performed a sensitivity analysis, replicating the principal models with these items removed (modified outcome score/24 points).

After estimating each model, we checked assumptions using plots of the standardized residuals. We performed all analyses using Stata v.17.0 (StataCorp, TX, USA).

### Data availability

Complete de-identified participant data, along with study protocols, and a variable dictionary, are available through the Dementias Platform UK Data Portal: https://portal.dementiasplatform.uk.

## Results

Of 1510 participants recruited, median age was 77 [interquartile range (IQR) 73 to 82], and 57% were female (Table 1). We undertook home assessments in *n* = 32 participants because hearing impairment precluded telephone interview. Over the study period (follow-up to July 2021), 209 participants (13.6%) were hospitalized across 371 episodes, with 1566 days of data collection, totalling 1999 person-days of assessment following imputation to account for weekends and bank holidays (Fig. 1). Elective admissions accounted for 6% episodes (22/371) and 88 bed days (4.4%). In emergency admissions, hospitalized individuals had lower baseline TICS-m cognitive scores (mean 35.5 versus 38.8 points, *P* < 0.01) and more frailty (frailty index 0.25 versus 0.15, *P* < 0.01) than those not hospitalized. Individuals admitted once accounted for 114 (55%) hospital episodes; the rest were admitted multiple times (median number recurrent admissions 2, IQR 2 to 4). The median time from baseline cognitive assessment to admission was 289 days (IQR 130 to 447 days).

**Table 1:** Characteristics of the cohort in relation to hospitalization and delirium status

	Whole cohort	Hospitalized		Delirium	
	*n* = 1511	*n* = 209	*P*	*n* = 115	*P*
**Whole cohort**					
Age	77.8 (6.2)	80.7(6.4)	<0.01	81.9 (6.6)	0.03
Female	57%	54%	0.56	55%	0.95
Education	–	–	<0.01	–	<0.01
ȃDegree level	65%	50%		40%	
ȃUp to secondary (12 y schooling)	21%	26%		30%	
ȃUp to primary (6 y schooling)	14%	24%		30%	
White ethnicity	94%	92%	0.45	89%	0.56
Frailty Index (SD)	0.15 (0.13)	0.25 (0.16)	<0.01	0.30 (0.17)	<0.01
TICS-m (total, SD)	38.8 (5.9)	35.5 (8.3)	<0.01	33.8 (8.7)	<0.01
Fluency (words, SD)	15.6 (6.2)	13.0 (7.0)	<0.01	11.6 (6.8)	<0.01
Fluency (animals, SD)	19.0 (7.0)	15.0 (7.8)		13.3 (7.4)	
Self-rated health (poor/very poor)	18%	42%	<0.01	49%	0.62
Past medical history					
ȃMyocardial infarction	21%	34%	<0.01	37%	0.86
ȃDiabetes mellitus	12%	19%	<0.01	19%	0.22
ȃHypertension	50%	62%	<0.01	61%	0.35
ȃStroke	9%	14%	<0.01	16%	0.11
ȃCancer	24%	28%	0.09	25%	0.13
ȃCOPD	14%	25%	<0.01	28%	0.75
Any impaired PADL	9%	23%	<0.01	31%	<0.01
ȃToileting	4%	5%	<0.01	7%	0.31
ȃDressing	4%	9%	<0.01	12%	0.17
ȃBathing	4%	11%	<0.01	16%	0.12
Any impaired IADL	73%	84%	<0.01	90%	<0.01
ȃShopping	18%	41%	<0.01	52%	0.05
ȃWalking outside	15%	34%	<0.01	43%	0.04
Length of stay (days, IQR)	–	2 (1–4)		4 (2–8)	<0.01
**Hospitalization**					
Presenting complaint (top five systems)					
ȃGeneral (malaise, fever)	–	14%		15%	0.48
ȃRespiratory (dyspnoea, cough)	–	14%		9%	0.03
ȃNeurological (delirium, weakness)	–	5%		9%	0.02
ȃCV (chest pain, palpitations)	–	6%		6%	0.99
ȃGI (abdominal pain, diarrhoea)	–	7%		5%	0.32
Sodium	–	137 (5.3)		139 (4.1)	<0.01
Potassium	–	4.2 (0.6)		4.4 (0.6)	<0.01
Creatinine	–	93.6 (66.3)		92.7 (52.3)	0.04
Haematocrit	–	0.34 (0.05)		0.36 (0.05)	<0.01
White cell count	–	9.7 (7.6)		9.2 (4.5)	0.61

Hospitalization = sample hospitalized at least once, *P*-values in hospitalized patients refers to comparison with whole cohort; *P*-values in patients with delirium refers to comparison with all hospitalized patients. Delirium = any occurrence of delirium during any admission, *P*-values refer to comparisons with hospitalized sample. COPD = chronic obstructive pulmonary disease; CV = cardiovascular; GI = gastrointestinal; IADL = instrumental activities of daily living; PADL = personal activities of daily living.

The commonest presenting symptoms were general malaise and fever (15%), respiratory (dyspnoea, cough, 9%) and neurological complaints (confusion, 9%) (Table 1). Patients with delirium were less likely to have a respiratory presentation. There were some small statistically significant absolute differences in initial laboratory values (sodium, potassium, creatinine) for delirium patients, but these were unlikely to be clinically relevant (Table 1).

### Delirium status

On any given day (point prevalence), 29% of all hospitalized participants fulfilled DSM-IV criteria for delirium. At any assessment, participants met DSM-IV criteria A, B and C 69, 68 and 41% of the time (Table 2). Over the course of an admission, delirium was ascertained in 45% of inpatients (prevalent delirium at admission in 35%, incident delirium developing after admission in 10%). The average number of days with delirium (consecutively positive assessments) was 3.9 days.

**Table 2:** Point prevalence of delirium features in hospitalized sample contributing to DSM-IV case ascertainment from 1999 inpatient assessments

Criterion A: disturbance of consciousness 69%		Criterion B: change in cognition and/or perception 68%		Criterion C: acute and fluctuating change 41%	
Item 1 ≥ 2: reduced level of consciousness	33%	Item 2 ≥ 2: disorientation (time/place questions 5/10 errors)	32%	Item 10 ≥ 3: sleep–wake cycle disturbance	17%
Item 4 ≥ 2: impaired digit span (5 forwards or 3 backwards errors)	10%	Item 3 ≥ 2: short-term memory impairment (≥2 errors on 3-item delayed recall)	31%	Observed fluctuations in arousal	6%
Item 5 ≥ 2: inattention	30%	Item 6 ≥ 2: disorganized thinking	15%	Observed motor fluctuations	5%
Inattention during interview	4%	Item 7 ≥ 2: perceptual disturbance	13%	Informant report of fluctuations	22%
Dozes off during interview	1%	Item 8 ≥ 2: delusions	25%	MDAS or OSLA score different from previous assessment by ≥1 SD	5%
Distracted by environmental stimuli	3%	Informant report ‘more confused’	7%	–	–
OSLA total ≥2	31%	'Odd thoughts' described on direct questioning	2%	–	–
MOTYB >5 mistakes	13%	Hallucinations described on direct questioning	3%	–	–
Serial 7 score lower than baseline	16%	'Strange things' described on direct questioning	1%	–	–
–	–	Three sentences to complete (three-choice answer) (any error)	8%	–	–
–	–	Two sentences to complete (free choice answer) (either error)	7%	–	–
–	–	Two-stage sequencing command (either error)	7%	–	–

Each MDAS item is rated 0, 1, 2 or 3. Criterion present if one or more symptom/sign positive. Note MDAS item 9 (decreased or increased psychomotor activity) is not used in the case definition. MOTYB = months of the year backwards; Informants = health care staff and/or family/carers.

Measures contributing to Criterion A included abnormal OSLA scores (31%) and inability to perform months of the year backwards (13%). In those able to undertake serial subtractions of 7 from 100 at baseline, 16% could not do so on hospitalization. Digit span was impaired in 10% of individuals.

Components of Criterion B included short-term memory impairment in 31% of cases and 32% could not answer at least 5/10 orientation questions correctly. Disorganized thinking was apparent in 15% of individuals. There was evidence of perceptual disturbance in 13%.

There was fluctuation (Criterion C) in OSLA or MDAS scores (differing from the previous assessment by ≥1 SD) 5% of the time. Informants (ward staff and/or visitors) described fluctuations in arousal or motor function in 22%. New severe sleep–wake cycle disturbance was present in 17%.

### Baseline cognition and delirium severity

**Figure 2:**
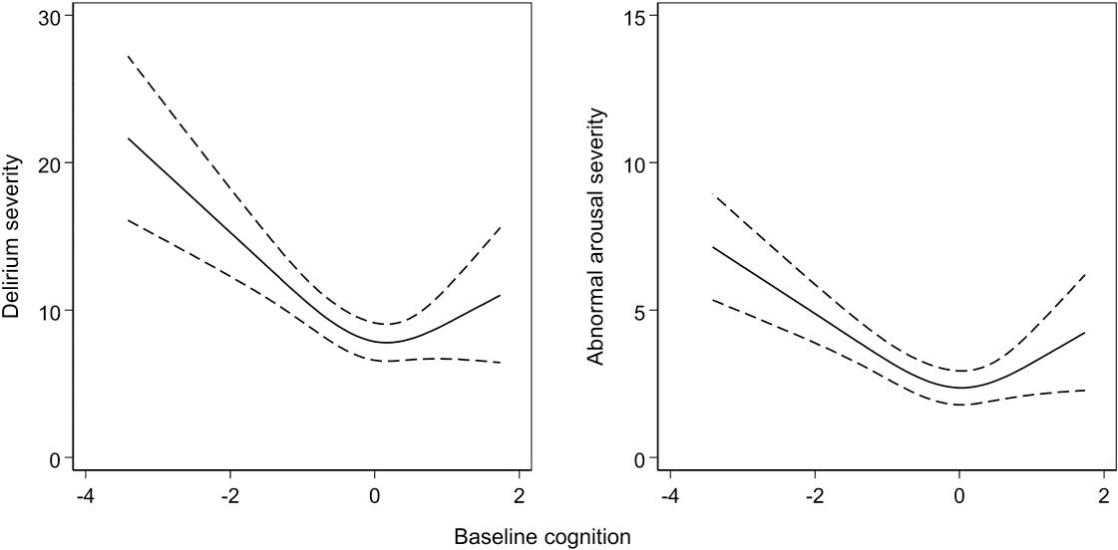
**Variation in delirium severity and abnormal arousal and baseline cognition.** *Left*: Delirium severity measured by MDAS scores. *Right*: Abnormal arousal severity measured by OSLA scores. Restricted cubic splines fitted across the range of baseline cognition (*z*-scores), defined by the TICS-m and augmented by two verbal fluency tasks.

Overall, there was a non-linear relationship between baseline cognition and delirium severity (Table 3 and Fig. 2). MDAS scores were higher when baseline cognition was both low and high. The negative relationship between baseline cognition and delirium severity for the first spline and positive relationship with the second spline led to MDAS scores of 14 (95% CI 10 to 19) points at *z*-score = −2 and MDAS of score 7.9, 95% CI 4.9 to 11 at *z*-score = +2) (Fig. 2). The lowest MDAS severity scores were seen in those at the midpoint of baseline cognition (*z*-score = 0).

**Figure 3:**
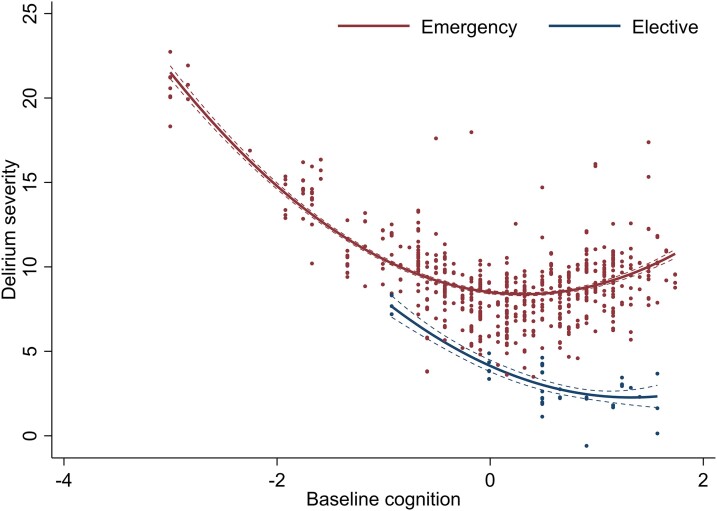
**Variation in delirium severity and baseline cognition, stratified by admission type.** Delirium severity measured by MDAS scores. Quadratic terms fitted across the range of baseline cognition (*z*-scores), defined by the TICS-m and augmented by two verbal fluency tasks.

**Table 3:** Delirium severity, by setting and/or possible aetiology, before and after adjustment by baseline cognition

	Adjustment per aetiology/setting				Multivariable adjustment			
	β	95% CI		*P*	β	95% CI		*P*
Cognition (first spline)	−5.00	−7.11	−2.89	<0.01	−4.56	−6.53	−2.59	<0.01
Cognition (second spline)	4.81	2.11	7.52	<0.01	4.34	1.73	6.96	<0.01
Elective admission	−4.83	−6.85	−2.81	<0.01	−2.95	−5.91	−0.34	0.03
Elective × cognition (second spline)	–	–	–		−2.10	−3.92	−0.27	0.03
Cognition (first spline)	−4.97	−7.07	−2.87	<0.01	–	–	–	
Cognition (second spline)	4.94	2.21	7.67	<0.01	–	–	–	
CRP ≥20 mg/l	1.33	−0.01	2.68	0.05	2.28	0.75	3.81	<0.01
Cognition (first spline)	−4.99	−7.08	−2.89	<0.01	–	–	–	
Cognition (second spline)	4.92	2.18	7.66	<0.01	–	–	–	
White cell count								
ȃ< 4 × 10^9^ cells/l	−3.10	−6.08	−0.12	0.04	−3.37	−6.74	−0.27	0.03
ȃ4–11 × 10^9^ cells/l	–	–			–	–	–	
ȃ≥11 × 10^9^ cells/l	−0.48	−1.36	0.41	0.29	−2.00	−4.00	−0.52	0.01
Cognition (first spline)	−4.87	−6.92	−2.82	<0.01	–	–	–	
Cognition (second spline)	4.77	2.08	7.45	<0.01	–	–	–	
Acute kidney injury	−1.35	−4.17	1.47	0.35	−1.89	−4.48	0.69	0.15
Cognition (first spline)	−4.88	−6.92	−2.85	<0.01	–	–	–	
Cognition (second spline)	4.73	2.01	7.45	<0.01	–	–	–	
Haemoglobin <100 g/l	0.61	−1.14	2.35	0.50	0.64	−1.38	2.66	0.53
Cognition (first spline)	−4.96	−6.94	−2.98	<0.01	–	–	–	
Cognition (second spline)	4.70	2.02	7.38	<0.01	–	–	–	
Sodium								
ȃ<125 mM/l	8.58	4.35	12.80	<0.01	8.72	4.48	13.0	<0.01
ȃ125–135 mM/l	1.31	−0.67	3.28	0.19	1.07	−0.84	2.97	0.27
ȃ135–145 mM/l	–	–	–		–	–	–	
ȃ≥145 mM/l	0.77	−0.14	1.68	0.10	1.49	0.03	2.95	0.05

Coefficients represent MDAS points (out of 30). First spline = restricted cubic spline describing first slope for lower cognition towards an inflection midpoint (knot). Second spline = restricted cubic spline describing second slope for higher cognition after an inflection midpoint (knot). All multivariable estimates also adjusted by age, sex, frailty index and NEWS (coefficients not shown). Acute kidney injury derived by algorithm from NHS England https://www.england.nhs.uk/wp-content/uploads/2014/06/psa-aki-alg.pdf. CRP = C-reactive protein.

Sensitivity analyses with disorientation and short-term memory items removed from the MDAS showed similar a similar bimodal distribution of scores (Supplementary Table 3 and Supplementary Fig. 3).

### Baseline cognition and abnormal arousal

The relationship between baseline cognition and abnormal arousal followed a comparable pattern. At the extremes of baseline cognition, OSLA scores were higher (OSLA 6.2, 95% CI 4.8 to 7.6 points at *z*-score = −2; OSLA 5.2, 95% CI 3.7 to 6.6 at *z*-score = + 2) (Fig. 2). Again, the lowest OSLA scores were recorded in those with baseline cognition *z*-scores of 0.

### Hospital setting and delirium severity

Elective admissions were associated with lower MDAS scores (Table 3). There was an interaction between higher baseline cognition (second spline) and elective status. This effect countered the positive base coefficient (*β* = 4.3, 95% CI 1.7 to 7.0) with negatively sloping estimates (elective *β* = −3.0, 95% CI −5.9 to −0.34; interaction *β* = −2.1, 95% CI −3.9 to −0.27) (Table 3). Together, this meant the relationship between baseline cognition and delirium severity was linear in elective, but not emergency settings (Fig. 3). Surgical admissions (regardless of elective or emergency status) were also associated with lower MDAS scores (−3.4 points, 95%CI −6.2 to −0.5, *P* = 0.02) (Supplementary Table 4). However, on further adjustment by elective or emergency setting, this association was no longer significant.

### Aetiology and delirium severity

In all cases, adjusting for possible aetiological precipitants derived from laboratory results contemporaneous with delirium assessments did not alter the underlying relationship between baseline cognition and MDAS scores (Table 3). In mutually adjusted models, CRP above ≥20 mg/l and severe hyponatraemia (Na < 125 mM/l) were associated with increased delirium severity. Lower and higher total white cell counts (outside the range 4–11 × 10^9^ cells/l) were associated with lower MDAS scores. Concurrent acute kidney injury or anaemia was not related to delirium severity (Table 3).

## Discussion

For the first time in a sample of unscheduled admissions, we showed that baseline cognition had a bimodal relationship with delirium severity and abnormal arousal, even after accounting for conventional physiological measures of illness severity, laboratory indicators of aetiologies and frailty. That is, emergency patients with both low and higher baseline cognition had a higher severity of delirium. This was not the case for the small number of elective admissions, where the more established linear relationship between baseline cognition and delirium severity was evident. Higher delirium severity scores in those with poorer baseline cognition were not confounded by pre-existing cognitive impairment. Delirium severity and abnormal arousal were closely related at all levels of cognition. Our results indicate that when acute illness is sufficient to lead to delirium, different factors may be at play across the range of baseline cognitive function. In the context of higher baseline cognition, the presence of delirium could be an important indicator of acute illness in older people, over and above physiological indices such as NEWS (insofar as NEWS may be specific in older people but not be sensitive), because delirium severity likely predicts worse outcomes.^33^

Our data should be interpreted in the context of some limitations. Despite comprehensive methods to identify hospitalized participants, there is inevitably a degree of selection bias that would have missed cases who developed delirium but remained in the community, and a small number of hospitalizations may have occurred in acute hospitals outside a participant’s usual residence. Although we had the advantage of frequent clinical assessments, we made assumptions about missing data on delirium status over weekends and public holidays. Our exploration of possible differences attributable to underlying aetiology was limited to major categories that could be readily operationalized from laboratory abnormalities. A more comprehensive approach is an area of ongoing analysis, which includes possible effects related to medication use and a more detailed assessment of the temporal relationships between each factor and their interactions. In common with other observational studies, residual confounding may affect some of the estimates. Nonetheless, the prospective capture of brain symptoms before and during acute illness allows for the most systematic mapping of baseline cognition, hospitalization and delirium in a population-representative sample to date.

In respect to other studies, in a cohort admitted to ICU, the IQCODE, a retrospective estimate of pre-morbid cognitive impairment, was linked to different delirium trajectories in critical illness: baseline cognitive impairment was associated with worsening delirium severity.^34^ As with elective surgical patients, however, the spectrum of pre-existing cognitive impairment was narrower compared with our data. In a study of general medical hospitalizations, a retrospective chart-based diagnosis of dementia was associated with a higher peak delirium severity score.^19^ The only other study to prospectively ascertain delirium in unselected hospitalizations, the Delirium and Cognitive Impact in Dementia study, found that lower baseline MMSE scores were associated with binary delirium risk; the relationship with severity was not assessed.^20^ There have not been previous reports linking high baseline cognition with more severe delirium or greater arousal abnormalities.^33,34^ Our findings in respect of aetiology are also broadly consistent with other studies examining the relative contributions of delirium precipitants on outcomes.^35,36^ Although all of the associations in our current study adjusted for acute illness severity, NEWS may be an insufficient measure in older people, at both the lowest and highest ends of the baseline cognitive spectrum. The idea that changes in behaviour and cognition, such as delirium itself, could be the sole or at least the predominant feature of acute illness has been observed in COVID-19, leading to the proposal that it be incorporated into the case definition for older adults.^37^ Work on clinical outcomes after delirium in people with different levels of baseline cognition will investigate the degree to which delirium is a better marker of acute illness compared with standard physiological metrics.

Overall, these data have several potential implications for clinical care. In people with delirium, early assessment of pre-delirium cognitive function, such as with IQCODE, could assist in identifying those at risk of severe delirium. This is important because severe delirium involves a higher risk of distress and future post-traumatic stress symptoms.^38^ In those with higher baseline cognition, recall of distressing delirium symptoms may be more likely, warranting consideration of follow-up. The novel observation that patients with higher baseline cognition tended to have more severe delirium could also prompt enhanced management given the relatively worse long-term cognitive outcomes for these patients.^7^ Abnormal arousal, commonly present in severe delirium, may also lead to more patient safety issues: longer length of stay, greater rehabilitation needs, reduced bulbar function and aspiration pneumonia and inpatient falls. For those with lower baseline cognition, family and carer education may mitigate this through better recognition of the specific links between abnormal arousal and delirium. For example, this could be a focus for patients recently diagnosed with dementia in the memory clinic. Such patients have a 50% risk of being admitted acutely in the next 12 months and public understanding of delirium is suboptimal.^39–41^

In conclusion, worse baseline cognition increases the risk of delirium. In patients who develop delirium, low and high baseline cognition are linked with a higher severity of delirium. The relationship between baseline cognition and delirium severity advocates for assessment of baseline cognition in patients with delirium, even if this must be retrospectively obtained using informant tools. Additionally, in patients with risk of severe delirium enhanced evaluation of causes and delirium symptoms such as distress may be warranted.

## Supplementary Material

awad062_Supplementary_Data

## References

[1] Gibb K , Seeley A, Quinn T, et al The consistent burden in published estimates of delirium occurrence in medical inpatients over four decades: A systematic review and meta-analysis study. Age Ageing. 2020;49:352–360. 32239173 10.1093/ageing/afaa040 PMC7187871

[2] Partridge JS , Martin FC, Harari D, Dhesi JK. The delirium experience: What is the effect on patients, relatives and staff and what can be done to modify this? Int J Geriatr Psychiatry. 2013;28:804–812. 23112139 10.1002/gps.3900

[3] Reston JT , Schoelles KM. In-facility delirium prevention programs as a patient safety strategy: A systematic review. Ann Intern Med. 2013;158(5 Pt 2):375–380. 23460093 10.7326/0003-4819-158-5-201303051-00003

[4] Wilson JE , Mart MF, Cunningham C, et al Delirium. Nat Rev Dis Primers. 2020;6:90. 33184265 10.1038/s41572-020-00223-4 PMC9012267

[5] Anand A , Cheng M, Ibitoye T, Maclullich AMJ, Vardy E. Positive scores on the 4AT delirium assessment tool at hospital admission are linked to mortality, length of stay and home time: Two-centre study of 82,770 emergency admissions. Age Ageing. 2022;51:afac051. 35292792 10.1093/ageing/afac051 PMC8923813

[6] Goldberg TE , Chen C, Wang Y, et al Association of delirium with long-term cognitive decline: A meta-analysis. JAMA Neurol. 2020;77:1373–1381. 32658246 10.1001/jamaneurol.2020.2273 PMC7358977

[7] Tsui A , Searle SD, Bowden H, et al The effect of baseline cognition and delirium on long-term cognitive impairment and mortality: A prospective population-based study. Lancet Healthy Longev. 2022;3:e232-e241. 35382093 10.1016/S2666-7568(22)00013-7 PMC7612581

[8] Whitby J , Nitchingham A, Caplan G, et al Persistent delirium in older hospital patients: an updated systematic review and meta-analysis. Delirium. Published online 9 August 2022. 10.56392/001c.36822 PMC7614331 36936539

[9] Lindroth H , Bratzke L, Purvis S, et al Systematic review of prediction models for delirium in the older adult inpatient. BMJ Open. 2018;8:e019223. 10.1136/bmjopen-2017-019223 PMC5931306 29705752

[10] Pendlebury ST . Screening for delirium in acute stroke. Stroke. 2021;52:479–481. 33380166 10.1161/STROKEAHA.120.033192

[11] Lindroth H , Bratzke L, Twadell S, et al Predicting postoperative delirium severity in older adults: The role of surgical risk and executive function. Int J Geriatr Psychiatry. 2019;34:1018–1028. 30907449 10.1002/gps.5104 PMC6579704

[12] Saczynski JS , Marcantonio ER, Quach L, et al Cognitive trajectories after postoperative delirium. N Engl J Med. 2012;367:30–39. 22762316 10.1056/NEJMoa1112923 PMC3433229

[13] Wu Y , Shi Z, Wang M, et al Different MMSE score is associated with postoperative delirium in young-old and old-old adults. PLoS ONE. 2015;10:e0139879. 26460750 10.1371/journal.pone.0139879 PMC4603675

[14] Ahmed S , Leurent B, Sampson EL. Risk factors for incident delirium among older people in acute hospital medical units: A systematic review and meta-analysis. Age Ageing. 2014;43:326–333. 24610863 10.1093/ageing/afu022 PMC4001175

[15] O’Regan NA , Fitzgerald J, Adamis D, Molloy DW, Meagher D, Timmons S. Predictors of delirium development in older medical inpatients: Readily identifiable factors at admission. Journal of Alzheimer’s Disease. 2018;64:775–785. 10.3233/JAD-180178 29966197

[16] Inouye SK , Viscoli CM, Horwitz RI, Hurst LD, Tinetti ME. A predictive model for delirium in hospitalized elderly medical patients based on admission characteristics. Ann Intern Med. 1993;119:474–481. 8357112 10.7326/0003-4819-119-6-199309150-00005

[17] Pendlebury ST , Lovett N, Smith SC, Cornish E, Mehta Z, Rothwell PM. Delirium risk stratification in consecutive unselected admissions to acute medicine: Validation of externally derived risk scores. Age Ageing. 2016;45:60–65. 26764396 10.1093/ageing/afv177 PMC4711661

[18] Lam CY , Tay L, Chan M, Ding YY, Chong MS. Prospective observational study of delirium recovery trajectories and associated short-term outcomes in older adults admitted to a specialized delirium unit. J Am Geriatr Soc. 2014;62:1649–1657. 25243679 10.1111/jgs.12995

[19] Hshieh TT , Fong TG, Schmitt EM, et al Does Alzheimer’s disease and related dementias modify delirium severity and hospital outcomes? J Am Geriatr Soc. 2020;68:1722–1730. 32255521 10.1111/jgs.16420 PMC7725352

[20] Richardson SJ , Davis DHJ, Stephan BCM, et al Recurrent delirium over 12 months predicts dementia: Results of the delirium and cognitive impact in dementia (DECIDE) study. Age Ageing. 2021;50:914–920. 33320945 10.1093/ageing/afaa244 PMC8099011

[21] Tieges Z , McGrath A, Hall RJ, Maclullich AM. Abnormal level of arousal as a predictor of delirium and inattention: An exploratory study. Am J Geriatr Psychiatry. 2013;21:1244–1253. 24080383 10.1016/j.jagp.2013.05.003

[22] Tieges Z , Quinn T, MacKenzie L, et al Association between components of the delirium syndrome and outcomes in hospitalised adults: A systematic review and meta-analysis. BMC Geriatr. 2021;21:162. 33673804 10.1186/s12877-021-02095-z PMC7934253

[23] Davis D , Richardson S, Hornby J, et al The delirium and population health informatics cohort study protocol: Ascertaining the determinants and outcomes from delirium in a whole population. BMC Geriatr. 2018;18:45. 29426299 10.1186/s12877-018-0742-2 PMC5807842

[24] Cook SE , Marsiske M, McCoy KJ. The use of the modified telephone interview for cognitive Status (TICS-M) in the detection of amnestic mild cognitive impairment. J Geriatr Psychiatry Neurol. 2009;22:103–109. 19417219 10.1177/0891988708328214 PMC2913129

[25] Hsieh S , Schubert S, Hoon C, Mioshi E, Hodges JR. Validation of the Addenbrooke’s Cognitive Examination III in frontotemporal dementia and Alzheimer’s disease. Disorders Geriatric Cognitive. 2013;36(3–4):242–250. 10.1159/000351671 23949210

[26] Searle SD , Mitnitski A, Gahbauer EA, Gill TM, Rockwood K. A standard procedure for creating a frailty index. BMC Geriatr. 2008;8:24. 18826625 10.1186/1471-2318-8-24 PMC2573877

[27] Breitbart W , Rosenfeld B, Roth A, Smith MJ, Cohen K, Passik S. The memorial delirium assessment scale. J Pain Symptom Manage. 1997;13:128–137. 9114631 10.1016/s0885-3924(96)00316-8

[28] MacKnight C , Rockwood K. A hierarchical assessment of balance and mobility. Age Ageing. 1995;24:126–130. 7793334 10.1093/ageing/24.2.126

[29] Smith GB , Prytherch DR, Meredith P, Schmidt PE, Featherstone PI. The ability of the National Early Warning Score (NEWS) to discriminate patients at risk of early cardiac arrest, unanticipated intensive care unit admission, and death. Resuscitation. 2013;84:465–470. 23295778 10.1016/j.resuscitation.2012.12.016

[30] Pimentel MAF , Redfern OC, Gerry S, et al A comparison of the ability of the National Early Warning Score and the National Early Warning Score 2 to identify patients at risk of in-hospital mortality: A multi-centre database study. Resuscitation. 2019;134:147–156. 30287355 10.1016/j.resuscitation.2018.09.026 PMC6995996

[31] Selby NM , Hill R, Fluck RJ. NHS England “think kidneys” AKI programme. Standardizing the early identification of acute kidney injury: The NHS England national patient safety alert. Nephron. 2015;131:113–117. 26351847 10.1159/000439146

[32] Newson RB . Sensible parameters for univariate and multivariate splines. Stata J. 2012;12:479–504.

[33] Rudolph JL , Jones RN, Grande LJ, et al Impaired executive function is associated with delirium after coronary artery bypass graft surgery. J Am Geriatr Soc. 2006;54:937–941. 16776789 10.1111/J.1532-5415.2006.00735.X PMC2398689

[34] Lindroth H , Khan BA, Carpenter JS, et al Delirium severity trajectories and outcomes in ICU patients. Defining a dynamic symptom phenotype. Ann Am Thorac Soc. 2020;17:1094–1103. 32383964 10.1513/AnnalsATS.201910-764OC PMC7462321

[35] Chalmers LA , Searle SD, Whitby J, Tsui A, Davis D. Do specific delirium aetiologies have different associations with death? A longitudinal cohort of hospitalised patients. Eur Geriatr Med. 2021;12:787–791. 33725336 10.1007/s41999-021-00474-8 PMC8322002

[36] Girard TD , Thompson JL, Pandharipande PP, et al Clinical phenotypes of delirium during critical illness and severity of subsequent long-term cognitive impairment: A prospective cohort study. Lancet Respir Med. 2018;6:213–222. 29508705 10.1016/S2213-2600(18)30062-6 PMC6709878

[37] Hunt C , Olcott F, Chan T, Williams G. Failing the frail: The need to broaden the COVID-19 case definition for geriatric patients. Clinical Medicine. 2021;21(Suppl 2):9–10. 34078676 10.7861/clinmed.21-2-s9

[38] Langan C , Sarode DP, Russ TC, Shenkin SD, Carson A, Maclullich AMJ. Psychiatric symptomatology after delirium: A systematic review. Psychogeriatrics. 2017;17:327–335. 28127828 10.1111/psyg.12240

[39] Sommerlad A , Perera G, Mueller C, et al Hospitalisation of people with dementia: Evidence from English electronic health records from 2008 to 2016. Eur J Epidemiol. 2019;34:567–577. 30649705 10.1007/s10654-019-00481-x PMC6497615

[40] Gibb K , Krywonos A, Shah R, Jha A, Davis D. What prompts patients to present with delirium? Eur Geriatr Med. 2021;12:643–651. 33544389 10.1007/s41999-020-00443-7 PMC8149353

[41] van Montfort SJT , van Dellen E, Wattel LL, et al Predisposition for delirium and EEG characteristics. Clin Neurophysiol. 2020;131:1051–1058. 32199395 10.1016/j.clinph.2020.01.023

