# Supplementary material for: Extremes of baseline cognitive function determine the severity of delirium: a population study
Source: Brain. 2023 Feb 28;146(5):2132–41. doi: 10.1093/brain/awad062 (PMC10151184; doi:10.1093/brain/awad062)

**Supplementary Table 1.** Associations between frailty and delirium, with example items removed.

The frailty index is, by design, composed of self-weighting constituent items. This is because redundancy inherent within humans who are not frail means that any illness, when severe enough to result in a health deficit, will affect mobility and physical function – also affecting additional illnesses that arise from comorbid disease or its treatment.

Univariate estimates for the association between delirium and frailty with and without two example items removed (*bathing disability* and *diabetes*): coefficients are very similar.

|  | | **OR** | **95% CI** | | **p** |
| --- | --- | --- | --- | --- | --- |
| Frailty index | | 6.0 | 1.1 | 31.5 | 0.036 |
|  | with bathing disability removed | 6.2 | 1.0 | 37.1 | 0.044 |
|  | with diabetes removed | 6.3 | 1.1 | 34.4 | 0.034 |

**Supplementary Table 2.** Relationship between baseline cognition and MDAS scores estimated using fractional polynomials.

|  | **MDAS** | | | |
| --- | --- | --- | --- | --- |
|  | **β** | **95% CI** | | **p** |
| Cognition (first order, x^2^) | -2.07 | -2.90 | -1.22 | <0.01 |
| Cognition (second order, x^3^) | 0.36 | 0.20 | 0.52 | <0.01 |
| Age (per SD) | 0.37 | -0.87 | 1.61 | 0.56 |
| Sex (women vs men) | -1.17 | -3.10 | 0.76 | 0.23 |
| Frailty index (per SD) | 0.48 | -0.55 | 1.51 | 0.36 |
| NEWS (per SD) | -0.17 | -0.79 | 0.44 | 0.57 |
| Fractional polynomial terms expressed as y = x^2^ + x^3^  MDAS Memorial Delirium Assessment Scale  NEWS National Early Warning Score | | | | |

**Supplementary Table 3.** Sensitivity analysis for delirium severity, without disorientation and short-term memory items.

|  | | **MDAS**  (without disorientation and short-term memory items) | | | |
| --- | --- | --- | --- | --- | --- |
|  | | **β** | **95% CI** | | **p** |
| Cognition (first spline) | | -2.47 | -3.39 | -1.55 | <0.01 |
| Cognition (second spline) | | 2.49 | 1.10 | 3.89 | <0.01 |
| Age (per SD) | | 0.32 | -0.54 | 1.19 | 0.46 |
| Sex (women vs men) | | -0.78 | -2.12 | 0.56 | 0.25 |
| Educational attainment | |  |  |  | 0.76 |
|  | Up to primary (6 years) | [Ref] |  |  |  |
|  | Up to secondary (12 years) | 0.38 | -1.42 | 2.18 |  |
|  | Degree or higher | 0.59 | -1.00 | 2.19 |  |
| Frailty index (per SD) | | 0.24 | -0.57 | 1.06 | 0.56 |
| NEWS (per SD) | | -0.13 | -0.53 | 0.27 | 0.53 |
| Time to first assessment (months) | | 0.09 | -0.02 | 0.20 | 0.10 |
| MDAS Memorial Delirium Assessment Scale  NEWS National Early Warning Score | | | | | |

**Supplementary Table 4**. Delirium severity, by setting, before and after adjustment by baseline cognition.

|  | **Adjustment per setting** | | | |  | **Multivariable adjustment** | | | |
| --- | --- | --- | --- | --- | --- | --- | --- | --- | --- |
|  | **β** | **95% CI** | | **p** |  | **β** | **95% CI** | | **p** |
| Cognition (first spline) | -5.17 | -7.43 | -2.91 | <0.01 |  | -5.08 | -7.28 | -2.88 | <0.01 |
| Cognition (second spline) | 5.03 | 2.23 | 7.83 | <0.01 |  | 4.89 | 2.13 | 7.65 | <0.01 |
|  |  |  |  |  |  |  |  |  |  |
| Surgical admission | -3.37 | -6.28 | -0.45 | 0.02 |  | -1.62 | -5.55 | 2.32 | 0.42 |
| Elective admission |  |  |  |  |  | -3.45 | -7.28 | 0.38 | 0.08 |
| Coefficients represent Memorial Delirium Assessment Scale points (out of 30).  All multivariable estimates also adjusted by age, sex, frailty index and NEWS (coefficients not shown)  Surgical admission to surgical ward, compared with admission to medical ward  Elective admission, compared with emergency admission | | | | | | | | | |

**Supplementary Figure 1.** Raw MDAS scores by tertiles of baseline cognition.

**Supplementary Figure 2.** The relationship between baseline cognition and delirium severity (MDAS scores) fitted using fractional polynomials (y = x^2^ + x^3^)


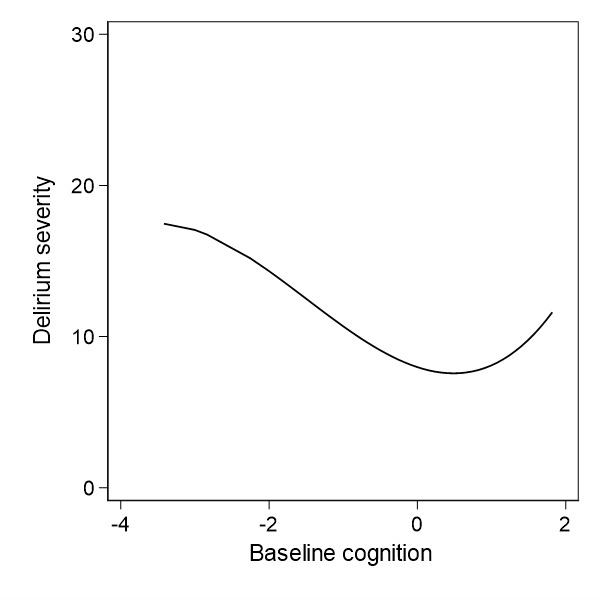


**Supplementary Figure 3.** Sensitivity analysis for delirium severity, without disorientation and short-term memory items.


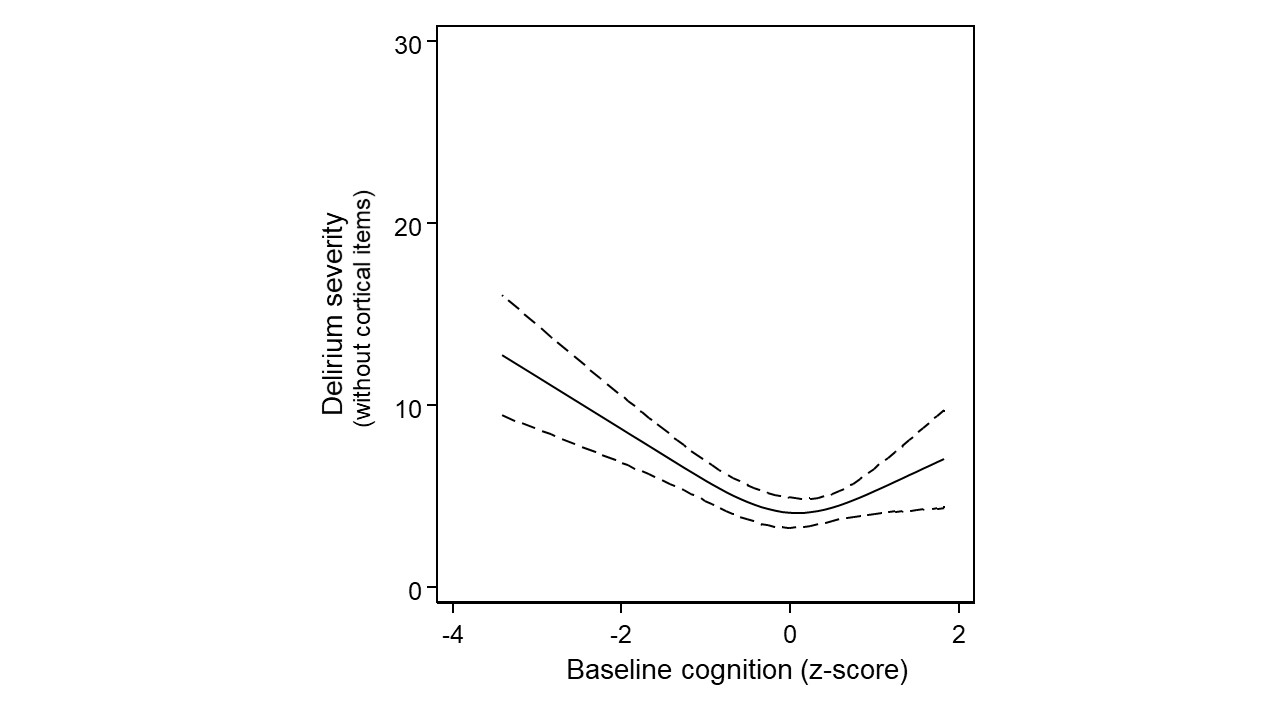

Supplement: awad062_Supplementary_Data [file awad062_supplementary_data.docx]
